# Mango performance as affected by the soil application of zeolite and biochar under water salinity stresses

**DOI:** 10.1007/s11356-022-21503-4

**Published:** 2022-07-08

**Authors:** Mohamed M. Harhash, Masoud M. M. Ahamed, Walid F. A. Mosa

**Affiliations:** Plant Production Department (Horticulture-Pomology), Faculty of Agriculture, Alexandria University, Saba Basha, Alexandria, 21531 Egypt

**Keywords:** Biochar, Zeolite, Mango, Fruit quality, Vegetative growth

## Abstract

This study was carried out during two consecutive seasons, 2020 and 2021, on 12-year-old mango (*Mangifera indica* L.). cv. Ewaise grown in region Idku, El Beheira Governorate, Egypt. The trees were planted at 5 × 4 m apart and grafted on “Sokary” root stock to study the influence of zeolite and biochar on growth, yield, and fruit quality of “Ewaise” mango cultivar irrigated by agricultural drainage water. The trees were treated by the following treatments: zeolite or biochar solely at 1, 2, and 3 kg for tree and their different combinations such as 1 kg zeolite + 1 kg biochar; 1 kg zeolite + 2 kg biochar; 1 kg zeolite + 3 kg biochar; 2 kg zeolite + 1 kg biochar; 2 kg zeolite + 2 kg biochar; 2 kg zeolite + 3 kg biochar; 3 kg zeolite + 1 kg biochar; 3 kg zeolite + 2 kg biochar; and 3 kg zeolite + 3 kg biochar as well as control zero soil application. The obtained results showed that the soil application of zeolite or biochar gave a positive effect on improving the soil characteristics which reflects on the tree trunk thickness, shoot length and thickness, number of inflorescences, yield in kg per tree, and fruit quality. The greatest positive effect on the previous mentioned parameters was obtained by the combined application of the soil application of 2 kg zeolite + 3 kg biochar; 2 kg zeolite + 2 kg biochar; 3 kg zeolite + 2 kg biochar; and 3 kg zeolite + 3 kg biochar over the rest-applied treatments or control in the two seasons.

## Introduction

Mango (*Mangifera indica* L.), which belongs to the Anacardiaceae plant family, is one of the most essential fruits in tropical and subtropical regions. Total cultivated area in Egypt was about 128,281 ha, which produced 1,395,244 tonnes, while the world production was 54,831,104 tonnes from the harvested area of 5,522,933 ha (FAO 2020).

Zeolite is characterized by a porous structure, where its pore is ranging from 0.3 to 1.0 nm. All zeolites work as screen for the particles, and they can absorb particles selectively depending on their size (Perez-Caballero et al. 2008). In addition to purifying water and wastewater, zeolite has also been used to remove ammonia and heavy metals (Wang and Peng 2009; Choudhary et al. 2022). Soil electrical conductivity, water absorption, and nutrient conservation can all be improved by using zeolite as a soil conditioner and because of its high water retention capacity (Islam et al. 2011; Sangeetha and Baskar 2016; Nakhli et al. 2017). It increased physical properties like permeability and moisture content and reduced soil erosion by reducing runoff and its rate during periods of drought or water stress, where the soil is becoming more humid, nutrient-depleted, and prone to erosion (Zahedi et al. 2011; Ghazavi 2015; Behzadfar et al. 2017). It can help in altering non-wetted sand and also aid in the distribution of water through the soil, as well as minimize the leaching of nitrate and raising the reservation of the soil nutrient (Szerment et al. 2014). Zeolite has a wide surface area that attracts microorganisms, and it is helpful in agricultural sector because of its great porous, cation exchange ability, and specificity for the cations of ammonium and potassium, as well as its capacity to work as a transporter for minerals (Smedt et al. 2015; Sangeetha and Baskar 2016). Furthermore, it plays a promising role in raising the efficiency usage of water, eliminating the negative pollutants from sandy soil, and improving the plant and the soil quality and consequently raising the yield of crops by less water usage (Ahmed et al. 2017).

Biochar is a sterile and odorless, and also it is high-carbon solid characterized by high levels from carbon and different effective groups that raise the soil water reservation ability (Downie et al. 2009; Atkinson et al. 2010; Busscher et al. 2010; Lehmann et al. 2011; Spokas et al. 2012; Basso et al. 2013; Clough et al. 2013). Guerena et al. (2013) reported that biochar could decrease the bioavailability of heavy metals and toxins, change the soil microorganisms’ distribution, and reduce the nutrients loss and the environmental pollution. Moreover, biochar can improve the soil physical properties like the soil porosity and water reservation, cation exchange capacity, and microorganism population and function. Additionally, it can also increase the elements and the soil moisture content, modifying the acidic soils and, therefore, consequently, the plant growth and the yield of the soil and the crops (Hardy et al. 2014; Gul et al. 2015; Gwenzi et al. 2015). Additionally, biochar is beneficial for increasing the growth and physiological and biochemical properties of the plant under saline conditions (Akhtar et al. 2015; Amini et al. 2016; Yang et al. 2015). Adding biochar to the saline soil increased the crop yield and productivity (Jeffery et al. 2015), and it raised the plant height and potassium uptake (Razaq et al. 2017). Besides, it is as a way to modify the nutrient cycle, reduce soil nitrous oxide emissions, and enhance carbon sequestration (Singh et al. 2015). Additionally, it is beneficial in reducing the impact of salt stress by enhancing the physical and chemical properties through sodium filtration and decreasing its concentration in the soil (Dahlawi et al. 2018). It could markedly affect the soil CO_2_ emissions (Oo et al. 2018) and improved the contaminated soil because of its high ability to absorb pollutants (She et al. 2018).

This experiment was done to investigate the role of biochar and zeolite as safe alternatives to the chemical fertilizers and in alleviating the undesirable impact of salinity stresses on vegetative growth, yield, and fruit quality of mango.

## Materials and methods

### Location and design

During 2020 and 2021 seasons, the present experiment on 12-year-old mango (*Mangifera indica* L.). cv. Ewaise grafted on “Sokary” root stock and grown at Idku, El Beheira Governorate, Egypt. At the distances of 5 × 4 m apart, the trees were cultivated. The analysis of the soil experiment was illustrated in Table 1.

**Table 1:** Physical and chemical properties for the soil of the experiment

Parameter	Sample		
Mechanical analysis		Macronutrients	
Soil depth	0–60 cm		
Sand	95.52%	N	132 ppm
Silt	%	P	12.0 ppm
Clay	4.48%	K	230 ppm
Textural class	Sand	Micronutrients	
pH	8.07–	Fe	5.41 ppm
EC	2.12 ds/m	Zn	8.12 ppm
Salinity	1356 ppm	Mn	0.95 ppm
Soluble cations		Cu	1.31 ppm
Na^+^	14.3 Meq/L	Heavy metals	
K^+^	0.9 Meq/L	Ni	0.91 ppm
Ca^+^	4.0 Meq/L	Cd	0.04 ppm
Mg^+^	2.0 Meq/L	Pb	3.87 ppm
		Cr	0.00 ppm
Soluble anions			
Cl^−^	12.5 Meq/L	CO_3_^2−^	0.0 Meq/L
HCO_3_^−^	6.0 Meq/L	SO_4_^2−^	2.7 Meq/L

### Analysis of water

Samples were taken from the water used for irrigation year, away from periods of rain (July) precipitation, in order to find out some of the chemical composition, as shown in Table 2.

**Table 2:** Water chemical composition of the used water in this study

Parameter	Sample		
Textural class		Macronutrients	
pH	7.46	NH_4_	0.32 mg/L
EC	3.23 ds/m	NO_3_	0.39 mg/L
Salinity	2067 ppm	P	0.7 mg/L
Soluble cations		Micronutrients	
Na^+^	462.5 ppm	Fe	0.39 mg/L
K^+^	46.4 ppm	Zn	0.02 mg/L
Ca^+^	140.0 ppm	Mn	0.03 mg/L
Mg^+^	48.0 ppm	Cu	0.14 mg/L
Soluble anions		Heavy metals	
Cl^−^	674.5 ppm	Ni	0.00 mg/L
HCO_3_^−^	427.0 ppm	Cd	0.00 mg/L
CO_3_^2−^	-	Pb	0.93 mg/L
SO_4_^2−^	302.4 ppm	Cr	0.00 mg/L

### Preparation of used materials

- Biochar was prepared by using rice husk as a raw material. Unique organic material is slow release uncoated fertilizers. Moreover, biochar can be used as a slow release uncoated fertilizer to increase the functionality of nitrogen fertilizers when added to sandy soils and reduce their environmental impacts (El Sharkawi et al. 2018) (Table 3).
- Zeolites can be described as materials made up of micro-aluminosilicate crystals, which are used as ion exchangers, in waste storage, in the handling of liquid waste, as separators in purification, and also in the surrounding treating (Xu et al. 2007). Zeolites are hydrated aluminosilicates of alkaline and alkaline earth element, which are present in over 50 and 150 natural and artificial shapes (Jha and Singh 2016) (Table 4).

**Table 3:** The chemical composition and some properties of the biochar used in the experiment

Parameter	Biochar	Unit
PH (1:10)	6.1	–
(1:10, water extract)		
EC soluble ions (1:10)	1.1	ds/m
Na	400	mg/kg
Nitrogen	50.86	mg/kg
Phosphorus	183.25	mg/kg
Potassium	2100	mg/kg
Total nutrients		
Nitrogen	0.2	%
Phosphorus	0.74	%
Potassium	1.8	%

**Table 4:** The chemical composition and some related properties of zeolite powder used in the experiment

Parameter		Zeolite	Unit
EC(1:5 water extract)		0.4	ds/m
Total nutrients			
Nitrogen		13.3	mg/kg
Phosphorus		10.5	mg/kg
Potassium		400	mg/kg
Chemical composition			
SiO_2_		68.15	%
Al_2_O_3_		12.3	%
Fe_2_O_3_		1.3	%
TiO_2_		0.2	%
CaO		3.95	%
MgO		0.9	%
Na_2_O		0.75	%
K_2_O		2.7	%
Ion exchange ability properties			
Total exchange	Ca^2−^	0.60–0.97	mol/kg
	K	0.24–0.47	mol/kg
	Mg^2+^	0.05–0.18	mol/kg
	Na^−^	0.01–0.17	mol/kg

### Experimental design

The current experiment comprised sixteen treatments, and each treatment was composed of six trees as replicates; thus, ninety-six trees were selected randomly in randomized complete block design as shown in Table 5.

**Table 5:** The applied treatments from zeolite and biochar and their combinations

Treatments	
T1	Control
T2	1 kg zeolite
T3	2 kg zeolite
T4	3 kg zeolite
T5	1 kg biochar
T6	2 kg biochar
T7	3 kg biochar
T8	1 kg zeolite + 1 kg biochar
T9	1 kg zeolite + 2 kg biochar
T10	1 kg zeolite + 3 kg biochar
T11	2 kg zeolite + 1 kg biochar
T12	2 kg zeolite + 2 kg biochar
T13	2 kg zeolite + 3 kg biochar
T14	3 kg zeolite + 1 kg biochar
T15	3 kg zeolite + 2 kg biochar
T16	3 kg zeolite + 3 kg biochar

The above-mentioned treatments were used to the soil of the trees at January 2020 and 2021 seasons to investigate their influence on the following:

### Vegetative growth

Four branches were marked on each side of every tree or replicate at the beginning of vegetative season, and then the vegetative growth parameters were measured such as trunk girth (cm), shoot length (cm), and shoot thickness (mm). Leaf area (cm^2^) was measured in the 1st week of September by taken thirty leaves by using the following equation (Demirsoy 2009; Abdelsalam et al. 2018):

$$LA=0.70\left(L\times W\right)-1.06$$

where *LA* = leaf area (cm^2^), *L* = maximum length of leaf (cm), and *W* = maximum width of leaf (cm).

During vegetative season, total chlorophyll was measured as a SPAD in the fresh leaves using Minolta chlorophyll meter (SPAD, 501).

### Fruit set and fruit drop percentages

On each replicate, the inflorescence number on each shoot was counted and recorded. Three weeks after flowering, the fruit set percentage was calculated according to this following equation:

$$\mathrm{Fruit}\;\mathrm{set}(\%)=\frac{No.of\;fruitlets}{No.of\;inflorescences}\times100$$

#### Fruit retention (%)

Sixty days after flowering, final fruit set percentage was calculated in the same sequence mentioned above for the fruit set percentage according to this equation:

$$\mathrm{Fruit}\;\mathrm{retention}(\%)=\frac{No.\;of\;preserved\;fruits}{No.\;of\;inflorescences}\times100$$

#### Fruit drop %

It was calculated as the difference where the fruit drop was during the period between the initial contracts until 60 days, and then, there is no fall until the final harvest:

$$\mathrm{Fruit}\;\mathrm{drop}\left(\%\right)=\mathrm{Fruit}\;\mathrm{set}-\mathrm{Fruit}\;\mathrm{retention}$$

### Yield per tree

The fruit yield was assessed on each replicate/tree resulting from the applied treatments as number of fruits/tree and weight of fruits in kg/tree.

### Physical fruit characteristics

To estimate the fruit physical characteristics, ten fruits from each tree were harvested during the time of maturity, where the fruits were in the yellow color stage and transported quickly to the laboratory in order to determine their fruit physical characteristics. Average of fruit weight was measured by weighting ten fruits from each tree and take their average. The average of fruit length is in cm, while the fruit diameter was measured by using a Digital Vernier Caliper (Suzhou Sunrix Precision Tools Co., Jiangsu, China). By weighting, the removed water after dipping fruits in the water, the fruit volume in cm^3^ was assessed. Also the peel, pulp, and seed weights were measured, and the then pulp and seed percentage was accounted as a percentage from weight of fruit. In the fresh fruit, the firmness was appreciated by using a Magness and Taylor pressure tester with a ^7^/_18_-inch plunger (mod. FT 02 (0–2 Lb., Via Reale, 63–48,011 Alfonsine, Italy) and expressed as (Ib/ Inch^2^).

### Chemical fruit characteristics

The percentage of total soluble solid percentage from fresh-cut mango (TSS %) was measured by a hand refractometer (ATAGO CO., LTD., Japan). Total acidity percentage was determined in fruit juice (AOAC 2005), where 5 ml from the obtained juice was used to determine titratable acidity percentage. It was expressed as grams of citric acid/100 ml fruit juice, then TSS/acid ratio was calculated. By the method of Nelson arsenate–molybdate colorimetric method (Nielsen 2010), total and reducing sugars were estimated calorimetrically. The difference between total sugars and reducing sugars is non-reducing sugar percentage. By the titration with 2,6-dichlorophenolindophenol (Nielsen 2017), juice content from vitamin C (ascorbic acid) was determined, while fruit carotene content was assessed as the method cited by Aquino et al. (2018).

### Leaf mineral content from macro- and micronutrients

Thirty leaf samples from different parts were taken monthly from the middle part of vegetative branch. The leaves were washed and dried for 72 h at 60 °C, and then the nutrient concentrations were analyzed as follows. Kjeldahl method was used to determine the N concentration in fruit leaves after sulfuric acid digestion. P was measured by colorimetry using a spectrophotometer. K, Mg, Ca, Zn, Mn, Pb, Ni, B, Mo, Cu, and Fe were measured by atomic absorption spectrophotometry (AAS) (Cruz et al. 2019).

### Statistical analysis

Data of the current study was statistically analyzed using MSTAT package and then subjected to analysis of variance (ANOVA), and means of treatments were compared using LSD at 0.05 according to (Ott and Longnecker 2015).

## Results

Results in Table 6 showed that the soil application of biochar and zeolite increased greatly the trunk thickness comparing with control in the two seasons. It was noticed that the most effective results were obtained by the soil application of T16, T15, T14, T13, and also by T12 over the other applied treatments during the study seasons. From the results, it could be concluded that the application of zeolite was more effective that the effect of biochar during our study. Concerning to shoot length, it was cleared that it was statistically improved by the soil application of zeolite and biochar over control in the two seasons. Moreover, the treatments of the soil application of zeolite combined with biochar were more efficient than the usage of each one solely. T16, T15, T14, and T13 gave the highest increments in shoot length more than the rest-applied treatments in the two seasons. In leaf area, the best results were obtained by T 16, T15, T13, and T14, as well as by T12 on the rest of the treatments applied in both seasons. Regarding shoot thickness, the addition of zeolite and biochar has a great influence on increasing the shoot thickness; T16, T15, T14, T13, and T12 gave the highest increment treatments in the two seasons. Moreover, the influence of zeolite was more effective than the influence of biochar, and the combination between them was higher than the sole application over control in the two seasons. Concerning to total chlorophyll, it was the soil application of zeolite and biochar comparing with control in the two seasons. Additionally, T16, T15, T13, and T14 gave the highest increments in total chlorophyll more than the rest-applied treatments in both seasons.

**Table 6:** Effect of zeolite and biochar on tree trunk thickness, shoot length, leaf area, shoot thickness, and total chlorophyll of “Ewaise” mango during 2020 and 2021 seasons

Treatments	Tree trunk thickness (cm)		Shoot length (cm)		Leaf area (cm^2^)		Shoot thickness (mm)		Total chlorophyll (SPAD)	
	2020	2021	2020	2021	2020	2021	2020	2021	2020	2021
T1	2.3^ k^	2.1^ l^	10.1^ m^	11.0^n^	101.1^ g^	101.5^j^	6.9^ l^	7.1^ l^	43.3^ g^	43.4^i^
T2	2.5^j^	2.7^ k^	11.8^j^	12.7^ l^	104.8^ef^	105.6^ h−j^	7.8^j^	8.1^j^	44.8^e−g^	45.5^hi^
T3	2.7^hi^	2.9^ij^	12.4^i^	13.7^j^	105.8^ef^	107.9^ g−i^	8.0^i^	8.3^ij^	45.8^ef^	47.3^f−h^
T4	2.9 fg	3.1^ fg^	13.1^ h^	15.1^i^	107.8^de^	110.5^ fg^	8.2^ h^	8.6^ h^	46.7^c−e^	48.1^ fg^
T5	2.5^j^	2.6^ k^	10.7^ l^	11.4^n^	103.1^ fg^	104.2^ij^	7.1^ l^	7.3^ l^	43.4^ g^	43.9^i^
T6	2.6^ij^	2.8^jk^	11.0^ l^	11.8^ m^	104.5^e−g^	105.2^ h−j^	7.5^ k^	7.8^ k^	44.3^ fg^	45.2^hi^
T7	2.7^hi^	2.9^ij^	11.4^ k^	13.2^ k^	105.4^ef^	108.1^ g−i^	7.7^j^	8.0^jk^	45.4^e−g^	46.8^gh^
T8	2.8^gh^	3.0^hi^	13.2^ h^	15.0^i^	107.2^de^	108.9^f−h^	8.3^ h^	8.5^hi^	45.3^e−g^	47.9^ fg^
T9	3.0^ef^	3.1^gh^	14.1^ g^	15.7^ h^	107.8^de^	110.6^ fg^	8.5^ g^	8.8^gh^	46.0^d−f^	47.9^ fg^
T10	3.1^de^	3.3^e^	15.6^e^	17.1^f^	110.8^d^	113.1^ef^	8.8^f^	9.2^ef^	48.0^ cd^	49.3ef
T11	2.9^ fg^	3.2^ef^	15.2^f^	16.5^ g^	114.9^c^	115.6^de^	8.6^ fg^	9.0^ fg^	48.3^c^	48.9^e−g^
T12	3.2^d^	3.5^d^	18.1^d^	19.6^e^	116.2^c^	118.4^ cd^	9.0^e^	9.4^e^	50.3^b^	51.0^de^
T13	3.4^c^	3.7^c^	20.4^c^	21.9^d^	117.7^c^	121.4^c^	9.6^c^	10.2^c^	51.3^b^	53.4^bc^
T14	3.5^c^	3.6^ cd^	20.1^c^	21.1^c^	116.5^c^	119.2^ cd^	9.2^d^	9.8^d^	50.4^b^	52.4^ cd^
T15	3.7^b^	4.0^b^	23.3^b^	25.2^b^	123.0^b^	127.7^b^	10.1^b^	11.0^b^	54.1^a^	55.1^b^
T16	4.1^a^	5.1^a^	25.6^a^	27.6^a^	133.2^a^	140.3^a^	11.4^a^	12.3^a^	55.5^a^	58.1^a^
LSD _0.05_	0.2	0.2	0.3	0.4	3.4	4.1	0.2	0.3	2.0	2.1

Means not sharing the same letter(s) within each column are significantly different at 0.05 level of probability

The data in Table 7 showed that the soil application of biochar and zeolite increased greatly the number of inflorescences; the soil application of zeolite combined with biochar was more effective than their separation, where T16, T15, T14, and T13 gave the highest increments in the inflorescence number more than the other treatments in the two seasons and control. Regarding to the fruit set, it was noticed that the most effective results were obtained by the soil application of T16, T15, T13, T14, and also by T12 over the rest-applied treatments in both seasons. Concerning to the fruit drop, the soil application of biochar and zeolite reduced the fruit drop percentage comparing with control in the two seasons. It was noticed that the most effective results were obtained by the soil application of T16, T15, T13, and T14 and by T12 over the rest-applied treatments in both seasons. Regarding to fruit retention, T16, T15, T13, and T14 gave the highest increments in fruit retention, more than the rest-applied treatments in both seasons. For the fruit number, it was noticed that it was improved by the soil application of zeolite and biochar comparing with control in the studying seasons. Moreover, T16, T15, T13, and T14 treatments gave the highest increments in the number of fruit more than the rest-applied treatments during experimental seasons. In the fruit yield, it was noticed that the soil application of zeolite and biochar was more effective than using each one of them alone. Generally, T16, T15, T13, and T14 gave the highest increments in yield more than the rest-applied treatments in both seasons.

**Table 7:** Effect of zeolite and biochar on inflorescence number, fruit set %, fruit drop %, fruit retention %, and number of fruit and yield of “Ewaise” mango during 2020 and 2021 seasons

Treatments	No. inflorescences/tree		Fruits set (%)		Fruit drop (%)		Fruit retention (%)		No. fruit/tree		Yield (kg/tree)	
	2020	2021	2020	2021	2020	2021	2020	2021	2020	2021	2020	2021
T1	195.0^ef^	197.0^e^	385.3^j^	385.4^ l^	312.9^a^	314.1^a^	72.5^ k^	71.3^n^	230^i^	239.0^ k^	59.4^ k^	62.3^j^
T2	194.0^f^	196.3^e^	391.0^gh^	392.3^ij^	311.5^ab^	305.7^ cd^	79.5^i^	86.1^jk^	240.0^gh^	249.0^ij^	63.3^ h−j^	66.3^ g−i^
T3	194.5^ef^	198.3^de^	392.2^ fg^	393.1^hi^	312.3^a^	306.2^c^	79.8^i^	86.9^j^	245.3^ fg^	253.3^ g−i^	66.1^f−h^	68.8^e−g^
T4	196.0^ef^	200.3^c−e^	392.5^ fg^	394.2^gh^	309.4^ cd^	301.6^ fg^	84.8^ fg^	92.6^ h^	258.3^e^	266.3^f^	71.0^de^	73.8^ cd^
T5	193.8^f^	198.3^de^	387.7^i^	389.6^ k^	312.0^ab^	310.7^b^	75.8^j^	79.0^ m^	234.8^hi^	245.8^j^	61.0^jk^	64.5^ij^
T6	194.3^ef^	198.3^de^	389.0^i^	390.6^jk^	312.1^a^	309.0^b^	76.7^j^	81.5^ l^	239.3^gh^	250.3^ h−j^	62.4^ij^	65.8^hi^
T7	195.5^ef^	200.0^c−e^	389.3^hi^	391.0^ij^	309.8^ cd^	305.4^ cd^	79.8^i^	85.6^ k^	245.0^ fg^	256.0^ g−i^	65.0^ g−i^	68.4^f−h^
T8	194.8^ef^	198.3^de^	393.7^f^	394.8^ fg^	311.1^ab^	304.0^de^	82.7^ h^	90.8^i^	250.3^f^	258.3^ g^	67.6^ fg^	70.2^ef^
T9	195.5^ef^	200.0^c−e^	394.0^f^	395.6^f^	310.6^b−d^	302.6^ef^	84.2^ g^	93.0^ h^	249.0^f^	257.0^gh^	68.6^ef^	71.5^de^
T10	197.8^de^	203.0^b−d^	396.5^e^	397.2^e^	310.9^b−d^	299.6^gh^	85.6^f^	97.6^f^	260.5^de^	269.5^ef^	73.2^d^	76.5^c^
T11	197.0^d−f^	200.5^c−e^	397.6^e^	397.8^e^	311.6^ab^	301.9^f^	85.8^f^	95.9^ g^	264.8^c−e^	273.8^de^	76.2^c^	79.5^b^
T12	199.5^ cd^	202.3^b−d^	398.6^d^	400.5^d^	307.0^ef^	298.4^hi^	91.6^d^	102.1^e^	266.5^ cd^	276.5^c−e^	76.8^c^	804^b^
T13	202.5^bc^	205.0^bc^	401.5^c^	404.5^c^	305.1^f^	296.7^i^	96.4^c^	107.8^c^	274.0b	284.0^ab^	79.8^b^	88.2^a^
T14	199.8^ cd^	203.0^b−d^	398.3^de^	402.9^c^	308.7^e^	297.0^i^	90.1^e^	105.9^d^	271.3^bc^	278.3^b−d^	84.3^a^	82.3^b^
T15	205.0^b^	207.0^b^	404.9^b^	408.7^b^	298.6^ g^	291.0^j^	106.4^b^	117.8^b^	276.5^b^	283.5^a−c^	85.9^a^	88.6^a^
T16	211.3^a^	218.3^a^	409.0^a^	415.5^a^	285.1^ h^	280.7^ k^	122.8^a^	134.8^a^	283.3^a^	290.3^a^	87.1^a^	89.9^a^
LSD_0.05_	3.1	4.4	2.1	2.2	2.3	2.1	1.2	1.2	6.7	6.7	2.8	2.3

Means not sharing the same letter(s) within each column are significantly different at 0.05 level of probability

The results in Table 8 showed that the soil application of biochar and zeolite increased greatly the fruit weight comparing with control in the two seasons. It was noticed that the most effective results were obtained by the soil application of T15, T13, T16, T14, and also by T12 over the rest-applied treatments in both seasons. Concerning to the fruit length, T16, T15, T14, and T13 gave the highest increments than the rest-applied treatments in the two seasons or control. Regarding to the fruit diameter, it was noticed that the most effective results were accompanied to the soil application of T16, T15, T13, T14, and also by T12 over the rest-applied treatments in both seasons. The fruit volume was increased by the application of T16, T15, T13, and T14 over the rest-applied treatments in both seasons.

**Table 8:** Effect of zeolite and biochar on fruit weight, fruit length, fruit diameter, and fruit volume of “Ewaise” mango during 2020 and 2021 seasons

Treatments	Fruit weight (g)		Fruit length (cm)		Fruit diameter (cm)		Fruit volume (cm^3^)	
	2020	2021	2020	2021	2020	2021	2020	2021
T1	258.3^f^	260.6^f^	10.2^j^	10.3^j^	6.8^ h^	7.0^ g^	214.0^ m^	217.5^ l^
T2	263.8^ef^	266.1^ef^	10.4^ij^	10.5^ij^	7.0^ fg^	7.2^ fg^	230.8^jk^	235.8^j^
T3	269.3^de^	271.8^de^	10.5^gh^	10.7^gh^	7.1^ef^	7.4^e^	234.0^j^	240.0^j^
T4	274.8^ cd^	277.3^ cd^	10.8^e^	11.0^e^	7.2^e^	7.5^de^	243.0^ h^	251.5^ g^
T5	260.0^f^	262.5^f^	10.2^j^	10.4^ij^	6.8^ h^	7.2^f^	219.0^ l^	226.0^ k^
T6	261.0^f^	262.8^f^	10.3^ij^	10.4^j^	6.9^gh^	7.3^ef^	227.8^ k^	236.3^j^
T7	265.3^ef^	267.1^ef^	10.4^hi^	10.6^hi^	6.9^gh^	7.3^ef^	233.0^j^	243.0^i^
T8	270.0^de^	271.8^de^	10.6^ fg^	10.8^ fg^	7.1^ef^	7.2^f^	238.0^i^	245.5^ h^
T9	275.5^ cd^	278.3^ cd^	10.7^ef^	10.9^ef^	7.1^ef^	7.3^ef^	243.0^ h^	253.0^ g^
T10	281.0^c^	283.8^c^	10.8^ef^	11.0^e^	7.2^e^	7.6^d^	255.0^f^	263.0^f^
T11	288.0^b^	290.6^b^	10.7^f^	10.9^e^	7.0^ fg^	7.3^ef^	251.0^ g^	262.3^f^
T12	288.3^b^	290.9^b^	11.1^d^	11.3^d^	7.4^d^	7.6^d^	271.0^e^	280.0^e^
T13	307.8^a^	310.4^a^	11.5^c^	11.7^c^	7.6^c^	7.9^c^	292.5^c^	311.8^c^
T14	294.0^b^	295.8^b^	11.1^d^	11.4^d^	7.4^d^	7.6^d^	281.0^d^	294.0^d^
T15	310.8^a^	312.5^a^	12.0^b^	12.4^b^	7.8^b^	8.3^b^	310.0^b^	320.3^b^
T16	307.5^a^	309.6^a^	13.2^a^	13.8^a^	9.1^a^	9.8^a^	325.5^a^	339.0^a^
LSD _0.05_	6.3	6.3	0.2	0.2	0.1	0.2	3.5	4.4

Means not sharing the same letter(s) within each column are significantly different at 0.05 level of probability

Data in Table 9 demonstrated that the fruit firmness was statistically improved by the soil application of zeolite and biochar comparing with control in the two seasons. Additionally, T16, T15, T14, and T13 gave the highest increments in fruit firmness in both two seasons compared to control. The best results in peel weight was improved by the soil application of zeolite and biochar comparing with control in the two seasons. Moreover, T16, T15, T14, and T13 gave the highest values in peel weight than the rest-applied treatments in during studying seasons or control. Concerning to pulp weight, it was noticed that the most effective results were obtained by the soil application of T16, T15, T13, and T14 as well as by T12 over the rest-applied treatments in both seasons. Seed weight was improved by the soil application of zeolite and biochar. Moreover, T16, T15, T13, and T12 gave the most significant increments in seed weight in both seasons and the control. Regarding to pulp, the best results were obtained by T16, T15, T13, and T14, as well as by T12 on the rest of the treatments applied in both seasons. The best results in seed and peel ratio were obtained by T16, T15, and T13 on the rest of the treatments applied in both seasons. Furthermore, combining between the soil application of zeolite and biochar was more effective than the application of each one individually.

**Table 9:** Effect of zeolite and biochar on fruit firmness, peel weight, pulp weight, seed weight, pulp %, seed–peel ratio of “Ewaise” mango during 2020 and 2021 seasons

Treatments	Fruit firmness (Lb/inch^2^)		Peel weight (g)		Pulp weight (g)		Seed weight (g)		Pulp (%)		Seed–peel ratio	
	2020	2021	2020	2021	2020	2021	2020	2021	2020	2021	2020	2021
T1	7.2^ m^	7.2^ l^	26.0^e^	26.8^ h^	177.0^p^	182.8^ k^	27.0^e^	27.5^f^	76.9^ g^	77.1^ k^	23.1^a^	22.8^a^
T2	7.8^ k^	8.0^ji^	27.0^de^	27.3^gh^	186.0^ m^	190.0^j^	27.0^e^	27.3^f^	77.5^ fg^	77.8^j^	22.5^b^	22.3^ab^
T3	8.2^j^	8.4^i^	27.0^de^	28.0^e−g^	190.0^ k^	198.3^i^	27.0^e^	28.5^ cd^	77.9^f^	78.1^hi^	22.1^de^	22.9^bc^
T4	8.9^ h^	9.3^ h^	27.0^de^	28.5^de^	196.0^i^	209.3^ g^	28.0^de^	29.5^b^	78.1^ef^	78.4^ fg^	22.1^e^	21.8^bc^
T5	7.3^ ml^	7.4^kl^	26.0^e^	27.0^gh^	182.0^o^	191.5^j^	27.0^e^	27.8^ef^	77.4^ fg^	77.7^j^	22.6^b^	22.2^ab^
T6	7.4^ l^	7.6^jk^	26.0^e^	27.0^gh^	184.0^n^	192.8^j^	27.0^e^	28.0^ef^	77.6^ fg^	77.8^ij^	22.4b^c^	22.2^ab^
T7	7.8^ k^	8.1^i^	26.0^e^	28.0^e−g^	188.0^ l^	201.3^ih^	28.0^de^	28.8^ cd^	77.7^ fg^	78.0^ij^	22.3^ cd^	22.1^ba^
T8	8.7^i^	9.0^ h^	26.8^e^	28.0^ fg^	193.0^j^	203.5^ h^	28.0^de^	28.5^de^	77.8^f^	78.2^gh^	22.2^c−e^	21.8^bc^
T9	9.5^ g^	9.9^ g^	27.0^de^	28.3^ef^	199.0^ h^	210.0^ g^	27.0^e^	28.5^c−e^	78.3^ef^	78.6^ef^	21.5^f^	21.3^bc^
T10	10.3^f^	10.6^f^	28.0^ cd^	29.3^ cd^	203.0^ g^	219.0^f^	29.0^ cd^	29.3^bc^	78.1^ef^	78.9^e^	21.9^e^	21.1^c^
T11	10.4^f^	10.7^f^	28.0^ cd^	30.0^bc^	211.0^f^	215.5^f^	29.0^ cd^	29.3^bc^	78.7^de^	78.8^e^	21.3^f^	21.2^bc^
T12	11.4^e^	11.7^e^	29.0^bc^	30.0^bc^	224.0^e^	235.3^e^	30.0^bc^	30.5^a^	79.1^d^	79.6^d^	20.6^ g^	20.4^ cd^
T13	12.4^c^	12.9^c^	29.8^ab^	31.3^a^	248.0^c^	261.3^c^	30.0^bc^	31.0^a^	80.5^bc^	80.8^c^	19.5^ h^	19.3^de^
T14	12.1^d^	12.5^d^	28.8^bc^	31.3^a^	241.0^d^	252.3^d^	29.8^bc^	30.5^a^	80.3^c^	80.5^c^	19.8^ h^	19.5^f^
T15	13.3^b^	14.0^b^	29.8^ab^	31.0^ba^	259.0^b^	273.5^b^	30.2^b^	30.9^a^	81.2^ab^	81.6^b^	19.1^i^	18.4^ef^
T16	14.4^a^	15.5^a^	30.5^a^	31.0^ba^	273.8^a^	294.3^a^	31.0^a^	32.0^a^	81.5^a^	82.7^a^	18.5^j^	17.5^f^
LSD _0.05_	0.2	0.4	1.0	1.1	1.8	3.7	1.1	1.0	0.8	0.4	0.3	0 .5

Means not sharing the same letter(s) within each column are significantly different at 0.05 level of probability

The data in Table 10 showed that TSS percentages, were statistically improved by the soil application of zeolite and biochar comparing with control in the two seasons. T16, T15, T13, and T14 gave the highest increments in both two seasons compared to control. Concerning to total sugar percentages were improved by the soil application of zeolite and biochar comparing with control in the two seasons. Moreover, T16, T15, T14, and T13 gave the highest increments in total sugar than the rest-applied treatments in the two seasons and control. Concerning to reducing sugar, it was noticed that the most effective results were accompanied with the soil application of T16, T15, T13, T14, and also by T12 over the rest-applied treatments in both seasons. Non-reducing sugar was improved by the soil application of zeolite and biochar. Moreover, T16, T15, T13, and T14 gave the highest increments in non-reducing sugar in both seasons and the control. Vitamin C was enhanced by the soil application of T16, T15, T13, and T14, as well as by T12 rather than the rest of the treatments in both seasons. Total acidity was reduced by T 16, T15, T13, and 14 in both seasons. Carotene content in the fruits was improved statistically by the soil application of zeolite and biochar. Moreover, T16, T15, T13, and T14 gave the highest increments in both two seasons compared to control.

**Table 10:** Effect of zeolite and biochar on “Ewaise” mango fruit content from TSS, Total, reduced, none reduced and fruit acidity percentages, vitamin C, and carotene during 2020 and 2021 seasons

Treatments	TSS (%)		Total sugars (%)		Reducing sugar(%)		Non-reducing sugars (%)		Total acidity (%)		Vitamin C (mg/100 ml juice)		Carotene mg/100 g	
	2020	2021	2020	2021	2020	2021	2020	2021	2020	2021	2020	2021	2020	2021
T1	18.5^n^	18.3^j^	10.8^ m^	10.7^ m^	4.2^ m^	4.4^ k^	6.7^ij^	6.3^j^	0.44^a^	0.43^a^	20.4^o^	20.0^p^	1.11^ k^	1.12^ k^
T2	20.3^ k^	20.5^ h^	11.4^ l^	11.6^ k^	4.6^jk^	5.0^i^	6.9^i^	6.6_i_	0.39^e^	0.38b^c^	23.4^kl^	23.7^ m^	1.17^hi^	1.21^i^
T3	21.4^j^	22.0^ g^	12.3^j^	12.5^j^	4.8^i^	5.3^gh^	7.5^ g^	7.2^ h^	0.38^ef^	0.37^ cd^	25.6^ k^	25.9^ k^	1.24^ g^	1.28^gh^
T4	22.6^ h^	23.3^f^	13.3^i^	13.9^i^	5.3^ h^	6.0^f^	8.0^f^	7.9^ g^	0.37^f^	0.36^d^	26.5^j^	27.0^j^	1.29^f^	1.36^f^
T5	18.9^ m^	19.1^j^	10.8^ m^	11.1^ l^	4.4^kl^	4.7^j^	6.4^ k^	6.4^ij^	0.43^b^	0.42^a^	21.3^n^	21.6^o^	1.12^jk^	1.15^jk^
T6	19.5^ l^	19.8^i^	10.9^ m^	11.5^ k^	4.4^ l^	4.9^ij^	6.6^jk^	6.6^i^	0.42^c^	0.41^a^	22.0^ m^	22.4^n^	1.13^jk^	1.18^ij^
T7	20.4^ k^	21.0^ h^	11.8^ k^	12.2^j^	4.6^j^	5.1^hi^	7.2^ h^	7.1^ h^	0.40^d^	0.39^b^	23.8^ l^	24.2^ l^	1.15^ij^	1.23^hi^
T8	21.8^i^	22.0^ g^	13.1^i^	13.6^i^	4.9^i^	5.4^ g^	8.2^f^	8.2^f^	0.37^f^	0.31^ fg^	27.0^i^	27.7^i^	1.20^ h^	1.23^hi^
T9	23.2^ g^	23.5^ef^	14.3^ h^	14.8^ h^	5.4^ h^	6.1^f^	8.9^e^	8.7^e^	0.35^ g^	0.34^e^	30.6^ h^	30.9^ h^	1.24^ g^	1.29^ g^
T10	24.4^e^	25.0^d^	15.4^f^	16.2^f^	5.8^f^	6.9^de^	9.6^d^	9.3^d^	0.34^ h^	0.31^ fg^	32.8^f^	33.7^f^	1.38^e^	1.49^e^
T11	23.8^f^	24.0^e^	14.8^ g^	15.3^ g^	5.6^ g^	5.9^f^	9.1^e^	9.4^d^	0.33^hi^	0.32^f^	31.9^ g^	32.4^ g^	1.32^f^	1.38^f^
T12	24.5^de^	25.3^d^	15.9^e^	16.6^e^	6.2^e^	6.7^e^	9.7^d^	9.9^c^	0.32^ij^	0.30^gh^	34.8^e^	35.3^e^	1.36^e^	1.54^e^
T13	25.3^c^	26.4^c^	17.0^c^	17.8^c^	6.7^c^	7.4^c^	10.3^b^	10.4^b^	0.31^j^	0.28^hi^	37.1^c^	38.4^c^	1.66^c^	2.16^c^
T14	24.6^d^	25.1^d^	16.5^d^	17.0^d^	6.6^d^	7.0^d^	10.0^c^	10.0^c^	0.32^ij^	0.31^ fg^	35.7^d^	37.0^d^	1.46^d^	1.92^d^
T15	26.0^b^	27.2^b^	17.7^b^	18.4^b^	7.2^b^	7.8^b^	10.5^ab^	10.6^b^	0.29^ k^	0.27^i^	38.8^b^	39.8^b^	2.12^b^	2.38^b^
T16	26.9^a^	28.5^a^	18.5^a^	19.8^a^	7.9^a^	8.6^a^	10.6^a^	11.2^a^	0.28^ l^	0.24^j^	42.7^a^	44.6^a^	2.75^a^	3.07^a^
LSD _0.05_	0.3	0.5	0.2	0.3	0.1	0.3	0.2	0.3	0.01	0.02	0.4	0.4	0.03	0.06

Means not sharing the same letter(s) within each column are significantly different at 0.05 level of probability

Results in Table 11 showed that the soil application of biochar and zeolite increased greatly the nitrogen content in the leaf comparing with control in the two seasons. It was noticed that the most effective results were obtained by the soil application of T16, T15, T14, T13, and also by T12 over the rest-applied treatments in both seasons. Regarding to leaf mineral content from phosphorous, it was observed that the most effective results were obtained by adding T16, T15, and T13 to the soil over the rest of the applied treatments in both seasons. For the potassium content in the leaves, it was improved by the soil application of zeolite and biochar. Moreover, T16, T15, T13, and T14 gave the highest increments in potassium in both seasons. Leaf lead content was decreased significantly by the soil application of T16, T15, T13, and T14 comparing with control or the rest-applied treatments in the two seasons. It was noticed that leaf content from nickel was minimized by the soil application of T16, T15, T13 and 14 over control in both seasons.

**Table 11:** Effect of zeolite and biochar on “Ewaise” mango leaf composition from nitrogen, phosphorous, potassium, lead, and nickel during 2020 and 2021

Treatments	N (%)		P (%)		K (%)		pb µg/mL		Ni µg/mL	
	2020	2021	2020	2021	2020	2021	2020	2021	2020	2021
T1	1.35^ l^	1.41^ m^	0.31^ k^	0.33^j^	1.10^ l^	1.04^ m^	3.02^a^	3.03^a^	0.44^a^	0.44^a^
T2	1.47^j^	1.56^j^	0.35^hi^	0.38^gh^	1.31^hi^	1.37^j^	2.51^b^	1.74^b^	0.22^c^	0.19^d^
T3	1.61^i^	1.72^i^	0.36^gh^	0.39^ fg^	1.36^ h^	1.42^i^	2.50^c^	1.64^c^	0.21^ cd^	0.18^de^
T4	1.74^ h^	1.83^ h^	0.37^ fg^	0.40^ fg^	1.42^ g^	1.50^ h^	2.12^e^	1.55^d^	0.18^ fg^	0.16^ fg^
T5	1.36^ l^	1.45^ l^	0.33^j^	0.35^ij^	1.09^ k^	1.16^ l^	2.22^d^	2.12^b^	0.29^b^	0.31^b^
T6	1.39^ k^	1.52^ k^	0.34^ij^	0.36^hi^	1.15^j^	1.22^ k^	1.90^ g^	1.69^e^	0.21^ cd^	0.26^c^
T7	1.48^j^	1.54^ k^	0.35^hi^	0.38^gh^	1.27^i^	1.35^j^	1.71^ h^	1.50^f^	0.19^d−f^	0.17^ef^
T8	1.73^ h^	1.84^ h^	0.36^gh^	0.40^ fg^	1.44^ g^	1.53^ h^	1.92^f^	1.53^e^	0.20^de^	0.18^de^
T9	1.88^ g^	1.93^ g^	0.37^ fg^	0.42^f^	1.49^f^	1.58^ g^	1.60^i^	1.33^ fg^	0.19^d−f^	0.16^ fg^
T10	2.04^f^	2.17^e^	0.39^de^	0.45^de^	1.56^e^	1.65^f^	1.42^ k^	1.22^ h^	0.17^gh^	0.15^ g^
T11	2.03^f^	2.12^f^	0.38^ef^	0.44^e^	1.53^ef^	1.60^ g^	1.51^j^	1.37^ g^	0.19^ef^	0.17^ef^
T12	2.21^e^	2.35^d^	0.40^ cd^	0.46^de^	1.72^d^	1.81^e^	1.35^ l^	0.95^ h^	0.18^ fg^	0.15^ g^
T13	2.31^c^	2.44^c^	0.41^bc^	0.49^bc^	1.99^b^	2.16^c^	1.31^n^	0.81^i^	0.16^hi^	0.13^ h^
T14	2.27^d^	2.37^d^	0.38^ef^	0.47^ cd^	1.93^c^	2.09^d^	1.32^ m^	0.91^ h^	0.17^gf^	0.15^ g^
T15	2.40^b^	2.54^b^	0.42^b^	0.50^b^	2.03^b^	2.22^b^	1.16^o^	0.69^i^	0.15^hi^	0.11^i^
T16	2.50^a^	2.71^a^	0.47^a^	0.61^a^	2.13^a^	2.31^a^	1.01^p^	0.36^j^	0.14^i^	0.08^j^
LSD _0.05_	0.02	0.03	0.17	0.02	0.05	0.03	0.01	0.21	0.02	0.01

Means not sharing the same letter(s) within each column are significantly different at 0.05 level of probability

Data in Table 12 cleared that the soil application of biochar and zeolite increased greatly iron content in the leaves comparing with control in the two seasons. It was noticed that the best results were obtained by the soil application of T16, T15, T14, and T13 in both seasons. Leaf zinc content was improved by the soil application of zeolite and biochar. T16, T15, T13, and T14 gave the highest increments from Zn in both seasons. Manganese content in the leaves was improved by the addition of T16, T15, and T13 comparing with control in both seasons. Copper content in the leaves was raised markedly by the soil application of T16, T15, T13, and T14 comparing with control in both seasons. Molybdenum content was raised greatly by the soil application of T16, T15, T13, and 14 over the rest of the treatments applied in both seasons. Boron was improved statistically by the soil amendment of zeolite and biochar. Additionally, T16, T15, T13, and T14 gave the higher increases over the rest of the treatments applied in both seasons.

**Table 12:** Effect of zeolite and biochar on “Ewaise” mango leaf composition from Fe, Zn, Mn, Cu, Mo, and B during 2020 and 2021 seasons

Treatments	Fe µg/mL		Zn µg/mL		Mn µg/mL		Cu µg/mL		Mo µg/mL		B µg/mL	
	2020	2021	2020	2021	2020	2021	2020	2021	2020	2021	2020	2021
T1	1.71^p^	1.83^o^	0.51^ l^	0.51^ m^	0.43^ k^	0.44^n^	0.60^ k^	0.62^o^	0.012^ k^	0.013^ k^	1.45^ m^	1.45^o^
T2	1.94^ m^	2.16^ l^	0.63^j^	0.65^ k^	0.47^j^	0.49^ l^	0.67^ij^	0.68^ m^	0.017^i^	0.021^ h^	2.12^i^	2.22^ k^
T3	2.06^ k^	2.20^ k^	0.66^i^	0.71^j^	0.50^i^	0.53^ k^	0.82^ h^	1.06^ k^	0.018^hi^	0.025^ g^	2.21^ h^	2.31^j^
T4	2.11^i^	2.37^j^	0.67^ h^	0.74^i^	0.57^ h^	0.59^i^	0.96^ g^	1.26^j^	0.022^ g^	0.029^f^	2.26^ g^	2.51^ g^
T5	1.85^o^	1.91^n^	0.62^ k^	0.64^ l^	0.45^jk^	0.46^ m^	0.61^ k^	0.63^o^	0.013^jk^	0.016^j^	1.66^ l^	1.83^n^
T6	1.91^n^	2.05^ m^	0.64^j^	0.65^ k^	0.46^j^	0.48^ l^	0.65^jk^	0.67^n^	0.014^j^	0.017^j^	1.99^ k^	2.07^ m^
T7	1.96^ l^	2.16^ l^	0.69^i^	0.70^j^	0.52^i^	0.54^ k^	0.71^i^	0.83^ l^	0.019^hi^	0.023^i^	2.08^j^	2.13^ l^
T8	2.09^j^	2.47^i^	0.69^ g^	0.74^hi^	0.65^ g^	0.58^j^	1.00^ g^	1.59^i^	0.018^i^	0.022^ h^	2.22^ h^	2.40^i^
T9	2.15^ h^	2.66^ h^	0.70^f^	0.75^gh^	0.69^f^	0.71^ h^	1.09^f^	1.65^ h^	0.019^ h^	0.024^ g^	2.34^f^	2.46^ h^
T10	2.22^f^	2.76^f^	0.71^e^	0.76^f^	0.74^d^	0.77^f^	1.26^e^	1.72^ g^	0.027^f^	0.031^f^	2.41^e^	2.61^e^
T11	2.19^ g^	2.72^ g^	0.71^f^	0.75^ fg^	0.72^e^	0.75^ g^	1.30^e^	1.90^f^	0.026^f^	0.027^e^	2.36^f^	2.58^f^
T12	2.33^e^	2.83^e^	0.72^e^	0.78^e^	0.72^de^	0.79^e^	1.69^d^	2.10^d^	0.028^e^	0.030^d^	2.43^d^	2.59^f^
T13	2.50^c^	3.04^c^	0.79^c^	0.87^c^	0.82^c^	0.85^c^	1.85^c^	2.52^c^	0.034^c^	0.037^c^	2.57^c^	3.03^c^
T14	2.42^d^	2.97^d^	0.75^d^	0.81^d^	0.80^c^	0.84^d^	1.71^d^	2.01^e^	0.030^d^	0.032^d^	2.55^c^	2.91^d^
T15	2.70^b^	3.12^b^	0.84^b^	0.91^b^	0.98^b^	1.10^b^	2.01^b^	3.17^b^	0.037^b^	0.041^b^	2.61^b^	3.10^b^
T16	2.82^a^	3.40^a^	0.85^a^	0.94^a^	1.02^a^	1.43^a^	2.46^a^	3.32^a^	0.041^a^	0.055^a^	2.92^a^	3.28^a^
LSD _0.05_	0.01	0.02	0.01	0.1	0.02	0.1	0.06	0.04	0.02	0.03	0.02	0.01

Means not sharing the same letter(s) within each column are significantly different at 0.05 level of probability

## Discussion

The results in our experiment proved that zeolite combined with biochar soil application has a crucial role in improving the shoot length, diameter, leaf area, leaf total chlorophyll, fruit set percentage, and fruit yield more the application of each one of them solely. Moreover, they also gave a beneficial effect in improving the fruit physical and chemical characteristics as well as the leaf miner al content from macro- and micronutrients comparing with control in the two seasons. These results were previously explained by the findings of many authors, they reported that zeolite is more effective in improving the efficiency of water use by increasing soil water holding capacity, and water availability to plants (Bigelow et al. 2004; Bernardi et al. (2010);, Shinde et al. (2010), and Colombani et al. (2015). Additionally, zeolite is essential for increasing crop yield (Noori et al. 2006) and works as stabilizer, a chelator, and fertilizer, where it can lose and gain water and helps in releasing the nutrients slowly (Perez-Caballero et al. 2008). In another study, the same authors reported that the addition of zeolite to olive soil increased the leaf content from nitrogen and potassium, water reservation, decreased the usage of fertilizers, and also the contamination of underground water. Additionally, the application of zeolite to strawberries and blackberries increased the yield, fruit chemical characteristics in terms of soluble solids and total acid contents (Glisic et al. 2009). The addition of grinded zeolite before planting, at 30, 45, and 60 t/ha. to apple increased soil nitrogen and potassium content by 2 to 3 times and improved also the sugar content, vitamin C, as well as the leaf and fruit mineral content from N, P, K, Ca, and Fe compared to untreated trees (Jakab and Jakab 2010). Milosevic et al. (2013) reported that the application of zeolite to “Roxana” apricot cultivar promoted tree growth, tree thickness, tree yield and yield efficiency, fruit weight, stone weight, flesh rate %, flesh firmness, soluble solids content, titratable acidity %, and ripening index as well as leaf N, P, K, Ca, Mg, Fe, Mn, Cu, Zn, and B compared to untreated trees. In addition, Santos et al. (2015) found the increase the content of pure sucrose and TSS of papaya fruits by adding different amounts of zeolite. Zeolites are able to adsorb CO_2_, which may influence photosynthesis, reduce leaf temperature by reflecting the infrared radiation, and consequently reduce transpiration rate, which may improve water use efficiency, yield, and the fruit quality (De Smedt et al. 2017). Using zeolite in the soil of bananas after harvest could delay the ripening of banana and improved its firmness and the peel color significantly (Tzeng et al. 2019). Application of zeolite to olive in silty clay soil increased shoot length, plant height, plant weight, number of branches, and number of leaves, trunk diameter and shoot diameter, leaf content from nitrogen and phosphorous, plant growth, and soil fertility as well as the relative water content (Al-Tabbal et al. 2020). Similarly, treating grape with zeolite showed increases in total soluble solids in grape (Calzarano et al. 2020). Additionally, the application of zeolite to *Carica papaya* L. cv. “Sekaki “ increased the availability and the absorption of N, P, and K, the plant growth, yield, and fruit quality, while it reduced the soil acidity (Choo et al. 2020). Mondal et al. (2021) reported that natural and surface-modified zeolite has selectivity for NH_4_ + , PO_4_^2−^, NO_3_ − , K + , and SO_4_^2−^ and reduces nutrient leaching. Moreover, they also stated that the unique characteristics of zeolites are helpful in improving the fertilizer and water use efficiency and, increasing the growth, yield and fruit quality of crops as well as it also has a desirable influence in reducing the environmental pollution via reducing nitrate leaching and the emissions of nitrous oxides and ammonia.

The addition of biochar on the orchard encouraged root development of apple (*Malus domestica Bork*) promoted the soil biological activity and soil respiration and raised the rates of nutrient cycling and the root development (Ventura et al. 2014). Applying biochar improved the trunk growth of apple (Eyles et al. 2015). In mango orchard, it was noticed that the application of biochar increased the fruit productivity by 16% (Van Vinh et al. 2015). Abo-Ogiala (2018) noticed that the addition of wood sawdust biochar at 0, 5, 10, and 20 Mg ha^−1^ in saline-sodic soil on “Grande Naine” banana increased the growth, productivity, and fruit quality in parallel to raising the rates of used biochar. Moreover, the same author noticed also that biochar increased the length and girth of pseudostem and leaf area, as well as bunch, cluster, and finger weights especially with 20 Mg ha^−1^. Moreover, the fruit quality parameters, i.e., number of fingers per cluster, finger length and diameter, pulp weight, peel weight, total soluble solids percentage, and total sugars and starch, as well as leaf mineral content significantly enhanced by increasing the application of biochar. In another study on “Volkamer” Lemon (*Citrus volkameriana*, Tenx pasq.) under saline condition, it was noticed that the application of biochar increased the leaf content from chlorophyll, N, P, and K^+^ comparing to untreated trees (Abo-Ogiala 2018). The soil addition of biochar to the seedlings of mango in the cultivation environment, which is composed from soil, sand, and biochar organic in the ratio of 2:1:1, raised the vigor, height, girth, of seedlings, and leaf number and leaf area when compared to other used media (Jasmitha et al. 2018). In the same trend, Naeem et al. (2018) found that the usage of biochar improved plant growth and the rate of photosynthetic and yield by improving the fruit retention. Suthar et al. (2018) stated that the addition of biochar improved the plant growth and fruit crop quality by the increasing the concentrations of NO_3_, P, Ca, and Mg and the fruit content from glucose, fructose, soluble solids, ascorbic acid, and sugar acid ratio over control in the two seasons. Besides, the addition of biochar to apple trees led to an increase by 37% and 300% from total organic carbon and available phosphorus, compared to control (Khorram et al. 2019; Kandil et al. 2020).

Kumari and Rajan (2019) reported that the addition of biochar improved significantly the growth performance of fruit trees, soil fertility like nutritional content, increased soil pH, cation exchange capacity, soil water holding capacity, optimized root system architecture, and decreasing soil bulk density. Additionally, in the same authors, it was noticed that the addition of biochar to mango, citrus, banana, and passion optimized water holding capacity, increased the yield, and improved the fruit quality. The fruit content from sugars was increased by the addition of biochar to the soil as a result of improving the nutrients and water absorption (Ali et al. 2017). Iqbal et al. (2013) reported that the addition of biochar at 20 and 40 mg/ha to the soil of mango cv. “Sufaid Chaunsa” significantly enhanced fruit retention, fruit weight, and consequently the fruit yield per plant compared to control. Additionally, it also improved the fruit content from total sugar content and TSS percentage, while it minimized the fruit acidity comparing with control in the two seasons. Our results also in the same trend with the findings of Moale et al. (2021); they reported that the application of zeolite on apricot and peach increased the yield, fruit quality, and water use efficiency up to 30%.

## Conclusion

The soil application of solely zeolite or solely biochar gave lower influence than that observed with the application of their combination. Also, the best treatments, which gave the highest increments in vegetative growth parameters, yield, and fruit quality in the two seasons, were the soil application of 3 kg zeolite + 3 kg biochar, 3 zeolite + 2 kg biochar, and 2 k zeolite + 2 kg biochar, respectively. Furthermore, the effect of zeolite was higher than the influence of biochar when each one of them was applied solely. Zeolite and biochar are considered as safe tools to improve the vegetative growth yield and fruit quality of fruit trees and thus can be applied as good alternatives to the chemical fertilizers.

## Data Availability

The data used to support the results of the present investigation are involved in the article.

## References

[CR1] Abo-Ogiala AMME (2018) Impact of biochar on growth, biochemical parameters and nutrients content of ‘volkamer’ lemon (*Citrus volkameriana**, **tenx* pasq.) under saline condition. Egyp J Hortic 45(2):305–314. 10.21608/ejoh.2018.4753.1073.

[CR2] Abdelsalam NR, Ali HM, Salem MZ, Ibrahem EG, Elshikh MS (2018) Genetic and morphological characterization of *Mangifera indica* L. growing in Egypt. Hort Sci 53(9):1266–1270. 10.21273/HORTSCI13084-18

[CR3] Ahmed TA, Al-Ghouti MA, Hussain N, Mahmoud AM (2017). An insights into growth characteristics of barely (*Hordeum vulgare*) in a zeolitic soil irrigated with saline water. Int J Agric Econ Devel.

[CR4] Akhtar SS, Andersen MN, Naveed M, Zahir ZA, Liu F (2015). Interactive effect of biochar and plant growth-promoting bacterial endophytes on ameliorating salinity stress in maize. Funct Plant Biol.

[CR5] Ali S, Rizwan M, Qayyum MF, Ok YS, Ibrahim M, Riaz M (2017). Biochar soil amendment on alleviation of drought and salt stress in plants: a critical review. Environ Sci Pollut Res.

[CR6] Al-Tabbal JA, Al-Mefleh NK, Al-Zboon KK, Tadros MJ (2020) Effects of volcanic zeolite tuff on olive (*Olea europaea* L.) growth and soil chemistry under a constant water level: five years ’monitoring experience. Environ Nat Resour J 18(1): 44–54. 10.32526/ennrj.18.1.2020.05.

[CR7] Amini S, Ghadiri H, Chen C, Marschner P (2016). Salt-affected soils, reclamation, carbon dynamics, and biochar: a review. J Soil Sediment.

[CR8] AOAC CA (2005) Official methods of analysis of the association of analytical chemists international. In: Official Methods Gaithersburg, MD. https://www.researchgate.net/ publication/292783651

[CR9] Aquino CF, Salomao LCC, Pinheiro-Santana HM, Ribeiro SMR, Siqueira DLD, Cecon PR (2018). Carotenoids in the pulp and peel of bananas from 15 cultivars in two ripening stages1. Rev Ceres.

[CR10] Atkinson CJ, Fitzgerald JD, Hipps NA (2010). Potential mechanisms for achieving agricultural benefits from biochar application to temperate soils: a review. Plant Soil.

[CR11] Basso AS, Miguez FE, Laird DA, Horton R, Westgate M (2013). Assessing potential of biochar for increasing water-holding capacity of sandy soils. Gcb Bioenergy.

[CR12] Behzadfar M, Sadeghi SH, Khanjani MJ, Hazbavi Z (2017). Effects of rates and time of zeolite application on controlling runoff generation and soil loss from a soil subjected to a freeze-thaw cycle. Int Soil Water Conserv Res.

[CR13] Bernardi AC, Monte MBDM, Paiva PRP, Werneck CG, Haim PG, Barros FDS (2010). Dry matter production and nutrient accumulation after successive crops of lettuce, tomato, rice, and andropogon grass in a substrate with zeolite. Rev Bras Cienc Solo.

[CR14] Bigelow CA, Bowman DC, Cassel DK (2004). Physical properties of three sand size classes amended with inorganic materials or sphagnum peat moss for putting green rootzones. Crop Sci.

[CR15] Busscher WJ, Novak JM, Evans DE, Watts DW, Niandou MAS, Ahmedna M (2010). Influence of pecan biochar on physical properties of a Norfolk loamy sand. Soil Sci.

[CR16] Calzarano F, Seghetti L, Pagnani G, Di Marco S (2020). Italian zeolitites in the control of grey mould and sour rot and their effect on leaf reflectance, grape and wine. Agri.

[CR17] Choo LNLK, Ahmed OH, Talib SAA, Ghani MZA, Sekot S (2020). Clinoptilolite zeolite on tropical peat soils nutrient, growth, fruit quality, and yield of *Carica papaya* L. CV Sekaki Agron.

[CR18] Choudhary RC, Bairwa HL, Kumar U, Javed T, Asad M, Lal K, Abdelsalam NR (2022). Influence of organic manures on soil nutrient content, microbial population, yield and quality parameters of pomegranate (Punica granatum L.) cv. Bhagwa. PloS one.

[CR19] Clough TJ, Condron LM, Kammann C, Muller C (2013). A review of biochar and soil nitrogen dynamics. Agron.

[CR20] Colombani N, Mastrocicco M, Di Giuseppe D, Faccini B, Coltorti M (2015). Batch and column experiments on nutrient leaching in soils amended with Italian natural zeolitites. CATENA.

[CR21] Cruz AF, Almeida GMD, Wadt PGS, Pires MDC, Ramos MLG (2019). Seasonal variation of plant mineral nutrition in fruit trees. Braz Arch Biol Technol.

[CR22] Dahlawi S, Naeem A, Rengel Z, Naidu R (2018). Biochar application for the remediation of salt-affected soils: challenges and opportunities. Sci Total Environ.

[CR23] De Smedt C, Steppe K, Spanoghe P (2017) Beneficial effects of zeolites on plant photosynthesis. Adv Mater Sci 2(1):1–11. 10.15761/AMS.1000115.

[CR24] Demirsoy H (2009). Leaf area estimation in some species of fruit tree by using models as a non-destructive method. Fruits.

[CR25] Downie A, Crosky A, Munroe P, Lehmann J, Joseph S (2009). Physical properties of biochar. Biochar for environmental management.

[CR26] El Sharkawi HM, Tojo S, Chosa T, Malhat FM, Youssef AM (2018). Biochar-ammonium phosphate as an uncoated-slow release fertilizer in sandy soil. Biomass Bioenergy.

[CR27] Eyles A, Bound SA, Oliver G, Corkrey R, Hardie M, Green S, Close DC (2015). Impact of biochar amendment on the growth, physiology and fruit of a young commercial apple orchard. Trees.

[CR28] FAO (2020) Food and agriculture organization of the United Nations. http://faostat-fao.org. 6086142

[CR29] Ghazavi R (2015). The application effects of natural zeolite on soil runoff, soil drainage and some chemical soil properties in arid land area. Int J Innov Appl Stu.

[CR30] Glisic IP, Milosevic TM, Glisic IS, Milosevic NT (2009). The effect of natural zeolites and organic fertilisers on the characteristics of degraded soils and yield of crops grown in western Serbia. Land Degrad Dev.

[CR31] Guerena D, Lehmann J, Hanley K, Enders A, Hyland C, Riha S (2013). Nitrogen dynamics following field application of biochar in a temperate north American maize-based production system. Plant Soil.

[CR32] Gul S, Whalen JK, Thomas BW, Sachdeva V, Deng H (2015). Physicochemical properties and microbial responses in biochar-amended soils: mechanisms and future directions. Agric Ecosyst Environ.

[CR33] Gwenzi W, Chaukura N, Mukome FND, Machado S, Nyama-soka B (2015). Biochar production and applications in sub-Saharan Africa: opportunities, constraints, risks and uncertainties. J Environ Manage.

[CR34] Hardy B, Dufey JE, Cornelis JT (2014) Former charcoal kiln sites where forest was cleared for cultivation: a case study of old biochar in cropland. EGU General Assembly Conferen Abstracts. 2561. https://orbi.uliege.be EGU 2014–2561.

[CR35] Iqbal J, Kiran S, Hussain S, Iqbal RK, Ghafoor U, Younis U, Zarei T, Naz M, Germi SG, Danish S, Ansari MJ, Datta R (2013). Acidified biochar confers improvement in quality and yield attributes of ‘sufaid chaunsa’ mango in saline soil. Hortic.

[CR36] Islam MR, Ren C, Zeng Z, Jia P, Eneji E, Hu Y (2011). Fertilizer use efficiency of drought-stressed oat (Avena sativa L.) following soil amendment with a water-saving superabsorbent polymer. Acta. Agric. Scand. Soil. Plant Sci.

[CR37] Jakab S, Jakab A (2010). Effects of the zeolitic tuff on the physical characteristics of haplic luvisol and the quality of fruits on apple orchards. Acta Univ Sapientiae Agric Environ.

[CR38] Jasmitha BG, Honnabyraiah MK, Anil Kumar S, Swamy GSK, Patil SV, Jayappa J (2018). Effect of enriched biochar on growth of mango seedlings in nursery. Int J Chem Stud.

[CR39] Jeffery S, Abalos D, Spokas KA, Verheijen FG (2015). Biochar effects on crop yield. Biochar for Environmental Management: Sci Technol Implem.

[CR40] Jha B, Singh DN (2016). Fly ash zeolites. Struct. Mater..

[CR41] Khorram MS, Zhang G, Fatemi A, Kiefer R, Maddah K, Baqar M, Zakaria MP, Li G (2019). Impact of biochar and compost amendment on quality, growth and yield of a replanted apple orchard in a 4-year field study. J Sci Food Agric.

[CR42] Kumari A, Rajan R (2019). Implication of biochar in fruit crop production. Marumegh.

[CR43] Lehmann J, Rillig MC, Thies J, Masiello CA, Hockaday WC, Crowley D (2011). Biochar effects on soil biota–a review. Soil Biol Biochem.

[CR44] Milosevic T, Milosevic N, Glisic I (2013). Tree growth, yield, fruit quality attributes and leaf nutrient content of ‘Roxana’ apricot as influenced by natural zeolite, organic and inorganic fertilisers. Sci Hortic.

[CR45] Moale C, Ghiurea M, Sirbu CE, Somoghi R, Cioroianu TM, Faraon VA, Lupu C, TricaB ADC, Oancea F (2021). Effects of siliceous natural nanomaterials applied in combination with foliar fertilizers on physiology, yield and fruit quality of the apricot and peach trees. J Plants.

[CR46] Mondal M, Biswas B, Garai S, Sarkar S, Banerjee H, Brahmachari K, Hossain A (2021). Zeolites enhance soil health, crop productivity and environmental safety. Agron.

[CR47] Naeem MA, Khalid M, Aon M, Abbas G, Amjad M, Murtaza B, Khan WUD, Ahmad N (2018). Combined application of biochar with compost and fertilizer improves soil properties and grain yield of maize. J Plant Nutr.

[CR48] Nakhli SAA, Delkash M, Bakhshayesh BE, Kazemian H (2017). Application of zeolites for sustainable agriculture: a review on water and nutrient retention. Water Air Soil Pollut.

[CR49] Nielsen SS (2010) Phenol-sulfuric acid method for total carbohydrates. In Food analysis laboratory manual. Springer: Boston, 47–53. DOI 10.1007/978-1-4419-1463-76.

[CR50] Nielsen SS (2017) Vitamin C determination by indophenol method. in food analysis laboratory manual. Springer, Cha 143–146. Doi: 10.1007/978-3-319-44127-6_15.

[CR51] Noori M, Zendehdel M, Ahmadi A (2006). Using natural zeolite for the improvement of soil salinity and crop yield. Environ Toxicol Chem.

[CR52] Oo AZ, Sudo S, Akiyama H, Win KT, Shibata A, Yamamoto A, Hirono Y, Sano T (2018). Effect of dolomite and biochar addition on N_2_O and CO_2_ emissions from acidic tea field soil. PLoS ONE.

[CR53] Ott RL, Longnecker MT (2015). An introduction to statistical methods and data analysis.

[CR54] Perez-Caballero R, Gil J, Benitez C, Gonzalez JL (2008). The effect of adding zeolite to soils in order to improve the NK nutrition of olive trees, preliminary results. Am J Agric Biol Sci.

[CR55] Razaq M, Shen HL, Sher H, Zhang P (2017). Influence of biochar and nitrogen on fine root morphology, physiology and chemistry of Acer mono. Sci Reports.

[CR56] Sangeetha C, Baskar P (2016) Zeolite and its potential uses in agriculture: a critical review. Agric Rev 37(2):101–108. 10.18805/ar.v0iof.9627.

[CR57] Santos EM, Cavakcante IHL, Silva JGB, Albano FG (2015). Impact of nitrogen and potassium nutrition on papaya ‘pawpaw’ fruit quality. Biosci J.

[CR58] She D, Sun X, Gamareldawla AH, Nazar EA, Hu W, Edith K (2018). Benefits of soil biochar amendments to tomato growth under saline water irrigation. Sci Reports.

[CR59] Shinde SA, Telang SM, Patil SS, Baig MMV (2010). Effect of clinoptilolite zeolite on mushroom growth. Asian. J Soil Sci.

[CR60] Singh R, Babu JN, Kumar R, Srivastava P, Singh P, Raghubanshi AS (2015). Multifaceted application of crop residue biochar as a tool for sustainable agriculture: an ecological perspective. Ecol Eng.

[CR61] Smedt CD, Someus E, Spanoghe P (2015). Potential and actual uses of zeolites in crop protection. Pest Manag Sci.

[CR62] Spokas KA, Cantrell KB, Novak JM, Archer DW, Ippolito JA, Collins HP, Nichols KA (2012). Biochar: a synthesis of its agronomic impact beyond carbon sequestration. J Environ Qual.

[CR63] Suthar RG, Wang CM, Nunes CN, Chen J, Sargent SA, Bucklin RA, Gao B (2018). Bamboo biochar pyrolyzed at low temperature improves tomato plant growth and fruit quality. Agric.

[CR64] Szerment J, Ambrozewich-Nita A, Kedziora K, Piasek J (2014) Use of zeolite in agriculture and environmental protection. A short review. UDC 666. 96;691.54. http://science.lpnu.ua/sites/default/ files/journal.

[CR65] Tzeng JH, Weng CH, Huang JW, Shiesh CC, Lin YH, Lin YT (2019). Application of palladium-modified zeolite for prolonging post-harvest shelf life of banana. J Sci Food Agric.

[CR66] Van Vinh N, Zafar M, Behera SK, Park HS (2015). Arsenic (III) removal from aqueous solution by raw and zinc-loaded pine cone biochar: equilibrium, kinetics, and thermodynamics studies. Int J Environ Sci Technol.

[CR67] Ventura M, Zhang C, Baldi E, Fornasier F, Sorrenti G, Panzacchi P, Tonon G (2014). Effect of biochar addition on soil respiration partitioning and root dynamics in an apple orchard. Euro J Soil Sci.

[CR68] Wang S, Peng Y (2009). Natural zeolites as effective adsorbents in water and wastewater treatment. Chem Eng J.

[CR69] Xu RWP, Yu Q, Huo J, Chen J (2007) Chemistry of zeolites and related porous materials: synthesis and structure Clementi Loop Singapore. John Wiley, 2(3):679–687. www.wiley.com

[CR70] Yang L, Liao F, Huang M, Yang L, Li Y (2015). Biochar improves sugarcane seedling root and soil properties under a pot experiment. Sugar Tech.

[CR71] Zahedi H, Rad AHS, Moghadam HRT (2011). Effects of zeolite and selenium applications on some agronomic traits of three canola cultivars under drought stress. Pesqui.

